# Prescriptions and Dispensing

**Published:** 1908-03-28

**Authors:** 


### THERAPEUTICS.

### PRESCRIPTIONS AND DISPENSING.

EMULSIONS (*concluded*).

THE use of an ordinary soap is naturally not resorted to for making emulsions for internal administration, except for veterinary purposes, and occasionally in other cases; for embrocations or liniments, however, soap is often a very useful agent, and soft soap, in which the alkali is potash, is usually employed for the purpose. The official liniment of turpentine is an illustration of this use of soap; the following simple turpentine emulsion is another example:—

℞ Saponis mollis ... ... ... ... ʒiv.

Olei Terebinthinæ ... ... ... ℥j.

Aquam ... ... ... ... ad ℥iv.

The soap is dissolved in an ounce of the water, previously heated, and the solution strained into the bottle; the turpentine is then added in small portions, and well shaken after each addition until it is all emulsified; the rest of the water is then added in the same way.

Various other emulsifying agents are in occasional use, although the very great majority of emulsions of all kinds are made with one or more of the agents that we have described. One of the chief of these subsidiary agents is *saponin*. This is a general name applied to a class of bodies occurring in many plants, some of them being highly poisonous. The solution of a small quantity of a saponin in water froths when shaken, like a soap solution, but even more readily. *Quillaia bark* is one of the commonest sources of saponin, and a tincture of this bark has strong emulsifying properties; for use for this purpose the tincture may be made of the strength of 4 oz. of the bark to the pint of rectified spirit. This tincture is serviceable for emulsifying many substances, the method of employment being simple; for example:—

℞ Olei ricini ... ... ... ... ... ℥j.

Tincturæ quillaiæ ... ... ... ... ʒj.

Aquam ... ... ... ... ad ℥ij.

Put the tincture into a bottle, add the oil, and shake. Then add the water, shake well, and the emulsion is made. On standing, the emulsified oil rises to the top in a sort of cream, which is readily diffused again on shaking.

℞ Extracti filicis liquidi ... ... ... ʒj.

Tincturæ quillaiæ ... ... ... ... ʒss.

Syrupi Zingiberis ... ... ... ... ʒss.

Aquam ad ℥j.

The method is similar to that just described.

Copaiba and turpentine may be emulsified by means of an equal quantity of tincture of quillaia.

Irish moss contains a considerable quantity of a mucilaginous constituent, which can be extracted by simply boiling with water. The decoction so obtained finds some application as an emulsifying agent for oils. The emulsions thus produced are generally characterised by considerable stability, but the oil is not as a rule so finely divided as it is by acacia mucilage. The following is an example of how it may be used:—

Irish moss ... ... ... ... ... ¼ oz.

Water ... ... ... ... ... 24 oz.

Soak together for an hour, boil for five minutes and strain.

℞ Cod-liver oil ... ... ... ... ℥j.

Irish moss mucilage ... ... ... ʒvj.

Water ... ... ... ... ... ... ʒij.

Misc.

A substance which has come into great favour during recent years is petroleum in the form of an emulsion. Sometimes the heavy petroleum oil is used, and sometimes the semi-solid soft paraffin, or vaseline. Petroleum cannot be emulsified by means of alkali, because it contains no fatty acids, and therefore cannot be saponified; but by the methods involving no chemical change, such as the use of a gum of some kind, or of yolk of egg, it can be treated like a vegetable or animal oil. If vaseline is used, it must first be melted, and emulsified at a temperature sufficiently high to prevent its resolidifying; hence a gum is preferable to an egg preparation in its case. The following is a typical formula:—

℞ Calcii hypophosphitis ... ... ... gr. xl.

Sodii hypophosphitis ... ... ... ʒj.

Pulveris acaciæ ... ... ... ... ℥j.

Paraffini liquidi ... ... ... ... ℥ij.

Elixir glusidi ... ... ... ... ♏xl.

Essentiæ olei amygdalæ ... ... ♏ss.

Aquam ... ... ... ... ad ℥vj.

The method to be followed in making the mixture up is the same as has been described for cod-liver oil.

Other solid substances of a fatty nature, such as oil of theobroma, spermaceti, and beeswax, are occasionally required in the form of emulsions. Generally speaking, the method to be followed in their cases is the same as for fixed oils, but the solid fat must first be melted and a hot pestle and mortar employed, so that solidification shall not occur until the emulsion is completed. The amount of gum acacia taken should be equal to that of the fat or wax to be dealt with.

*Essential oils* usually appear in emulsions only as flavouring agents, along with fixed oils. In such cases, if an alcoholic liquid is present, the essential oil may be dissolved in it; or, better still, it may be added to the fixed oil. If, however, an essential oil is to be emulsified by itself, mucilage is required in considerable quantity, and the mixing is best carried out in the bottle. Turpentine, more correctly termed oil of turpentine (*oleum terebinthinæ*), is an essential oil, and the directions we have given for dealing with it may be followed in other cases, though a larger proportion of gum will usually be required.

Terebene is of a similar nature, and should be treated in a similar manner.

We will now leave mixtures, and in our next article will begin to consider the prescribing and dispensing of pills. It is only possible to give an occasional illustrative case, but, as we have said, the main thing is to have a clear idea of the principles underlying dispensing, and therefore prescribing also. These principles we have entered into as fully as space would allow; and we would repeat our advice to him who is a novice in a dispensing practice—experiment with all sorts of prescriptions.

